# Meteorological Observations for March, 1857, Atlanta, Ga.

**Published:** 1857-04

**Authors:** J. G. Westmoreland


### METEOROLOGICAL OBSERVATIONS FOR FEBRUARY, 1857, AT ATLANTA, GA.

| MARCH. | THERMOMETER. | | | BAROMETER. | | | WIND. | REMARKS. |
|---|---|---|---|---|---|---|---|---|
| | 7 A. M. | 2 P. M. | 7 P. M. | 7 A. M. | 2 P. M. | 7 P. M. | | |
| 1 | 42 | 60 | 50 | 29.50 | 29.55 | 29.75 | W. | Windy—Fair. |
| 2 | 24 | 36 | 24 | 29.55 | 29.62 | 29.55 | W. | Windy—Fair. |
| 3 | 18 | 43 | 26 | 29.57 | 29.60 | 29.55 | W. | Hazy. |
| 4 | 25 | 60 | 40 | 29.50 | 29.62 | 29.55 | S. E. | Hazy. |
| 5 | 38 | 44 | 40 | 29.42 | 29.37 | 29.37 | W. | Cloudy—Rain $\frac{5}{8}$ in. |
| 6 | 25 | 38 | 28 | 29.50 | 29.60 | 29.65 | N. E. | Fair. |
| 7 | 20 | 31 | 29 | 29.67 | 29.77 | 29.72 | N. W. | Fair. |
| 8 | 26 | 44 | 37 | 29.70 | 29.77 | 29.67 | N. W. | Fair. |
| 9 | 34 | 40 | 34 | 29.57 | 29.55 | 29.57 | S. E. | Cloudy—Rain 1-16 in. |
| 10 | 20 | 45 | 40 | 29.51 | 29.65 | 29.55 | S. E. | Fair. |
| 11 | 34 | 39 | 38 | 29.47 | 29.50 | 29.57 | W. | Cloudy—Rain $\frac{3}{8}$ in. |
| 12 | 20 | 43 | 36 | 29.65 | 29.82 | 29.80 | N. W. | Fair. |
| 13 | 30 | 28 | 28 | 29.75 | 29.65 | 29.57 | E. | Cloudy, Sleet, R'n $\frac{1}{2}$ in. |
| 14 | 28 | 38 | 30 | 29.57 | 29.72 | 29.72 | E. | Cloudy. |
| 15 | 32 | 54 | 46 | 29.72 | 29.72 | 29.70 | S. | Hazy. |
| 16 | 30 | 50 | 42 | 29.65 | 29.67 | 29.70 | S. W. | Fair. |
| 17 | 36 | 64 | 50 | 29.65 | 29.72 | 29.72 | W. | Fair. |
| 18 | 48 | 52 | 46 | 29.67 | 29.53 | 29.53 | W. | Cloudy—Rain $\frac{1}{4}$ in. |
| 19 | 28 | 44 | 38 | 29.55 | 29.60 | 29.57 | N. W. | Fair. |
| 20 | 36 | 64 | 54 | 29.60 | 29.77 | 29.67 | S. W. | Fair. |
| 21 | 50 | 64 | 48 | 29.77 | 29.88 | 29.82 | S. W. | Fair. |
| 22 | 50 | 64 | 56 | 29.90 | 30 | 29.92 | W. | Fair. |
| 23 | 54 | 70 | 62 | 29.92 | 30 | 29.90 | N. | Hazy. |
| 24 | 50 | 76 | 60 | 29.82 | 29.92 | 29.77 | S. E. | Fair. |
| 25 | 50 | 64 | 52 | 29.80 | 29.82 | 29.67 | S. | Hazy. |
| 26 | 40 | 60 | 54 | 29.67 | 29.75 | 29.65 | W. | Fair. |
| 27 | 54 | 66 | 54 | 29.65 | 29.70 | 29.62 | W. | Fair. |
| 28 | 44 | 64 | 48 | 29.55 | 29.75 | 29.62 | S. W. | Hazy. |
| 29 | 40 | 62 | 52 | 29.65 | 29.77 | 29.72 | N. W. | Fair. |
| 30 | 36 | 58 | 46 | 29.72 | 29.90 | 29.82 | N. W. | Fair. |
| 31 | 40 | 64 | 52 | 29.85 | 29.87 | 29.72 | N. E. | Fair. |

Furnished by

J. G. WESTMORELAND, M. D.

